# Mobile Health Applications for the Most Prevalent Conditions by the World Health Organization: Review and Analysis

**DOI:** 10.2196/jmir.2600

**Published:** 2013-06-14

**Authors:** Borja Martínez-Pérez, Isabel de la Torre-Díez, Miguel López-Coronado

**Affiliations:** ^1^University of ValladolidDepartment of Signal Theory and Communications, and Telematics Engineering.University of ValladolidValladolidSpain

**Keywords:** apps, mHealth, mobile applications, prevalent conditions, World Health Organization (WHO)

## Abstract

**Background:**

New possibilities for mHealth have arisen by means of the latest advances in mobile communications and technologies. With more than 1 billion smartphones and 100 million tablets around the world, these devices can be a valuable tool in health care management. Every aid for health care is welcome and necessary as shown by the more than 50 million estimated deaths caused by illnesses or health conditions in 2008. Some of these conditions have additional importance depending on their prevalence.

**Objective:**

To study the existing applications for mobile devices exclusively dedicated to the eight most prevalent health conditions by the latest update (2004) of the Global Burden of Disease (GBD) of the World Health Organization (WHO): iron-deficiency anemia, hearing loss, migraine, low vision, asthma, diabetes mellitus, osteoarthritis (OA), and unipolar depressive disorders.

**Methods:**

Two reviews have been carried out. The first one is a review of mobile applications in published articles retrieved from the following systems: IEEE Xplore, Scopus, ScienceDirect, Web of Knowledge, and PubMed. The second review is carried out by searching the most important commercial app stores: Google play, iTunes, BlackBerry World, Windows Phone Apps+Games, and Nokia's Ovi store. Finally, two applications for each condition, one for each review, were selected for an in-depth analysis.

**Results:**

Search queries up to April 2013 located 247 papers and more than 3673 apps related to the most prevalent conditions. The conditions in descending order by the number of applications found in literature are diabetes, asthma, depression, hearing loss, low vision, OA, anemia, and migraine. However when ordered by the number of commercial apps found, the list is diabetes, depression, migraine, asthma, low vision, hearing loss, OA, and anemia. Excluding OA from the former list, the four most prevalent conditions have fewer apps and research than the final four. Several results are extracted from the in-depth analysis: most of the apps are designed for monitoring, assisting, or informing about the condition. Typically an Internet connection is not required, and most of the apps are aimed for the general public and for nonclinical use. The preferred type of data visualization is text followed by charts and pictures. Assistive and monitoring apps are shown to be frequently used, whereas informative and educational apps are only occasionally used.

**Conclusions:**

Distribution of work on mobile applications is not equal for the eight most prevalent conditions. Whereas some conditions such as diabetes and depression have an overwhelming number of apps and research, there is a lack of apps related to other conditions, such as anemia, hearing loss, or low vision, which must be filled.

## Introduction

Since the creation of the Internet, its massive use, especially in developed countries, has generated new forms of technology in almost every aspect of life [1]. One of these aspects is health care; Internet technologies have initiated major advances in telemedicine and telehealth, now present in every modern health care organization [2]. In the field of telehealth, eHealth has arisen as a paradigm involving the concepts of health, technology, and commerce, with commerce and technology as tools in the service of health [3]. Chang Liu et al (2011) perceive eHealth applications as the software applications that provide tools, processes, and communications in order to support electronic health care practice [4]. In addition to this, with the advent of wireless communications, there are no longer barriers of space and time between health care providers and patients [5]. The use of new wireless communications technology, such as mobile telecommunications networks (2.5G, 3G, 4G, HSPA+), Wireless Local Area Networks (WLAN), Wireless Personal Area Networks (WPAN) including Bluetooth and ZigBee, Wireless Body Area Network (WBAN), Wireless Sensor Networks (WSN), Radio-frequency Identification (RFID), and Worldwide Interoperability for Microwave Access (WiMAX), has greatly boosted telemedicine and eHealth [5-12].

In this context and thanks to these advances in communications, a new term arises: mHealth, a component of eHealth. The Global Observatory for eHealth (GOe) of the World Health Organization (WHO) defines mHealth or mobile health as “medical and public health practice supported by mobile devices, such as mobile phones, patient monitoring devices, personal digital assistants (PDAs), and other wireless devices” [13]. While new wireless technologies were being developed, new mobile devices were being created. In this way, PDAs, tablets, and smartphones appeared on the market. Although PDAs experienced a boom in the 1990s and early 2000s, they have been replaced by smartphones and tablets with new functions and utilities, which are common now in developed countries [4]. There are already more than 1.08 billion smartphones of a total of 5 billion mobile phones around the world, with 80% of the population having a mobile phone [14]. Regarding tablets, International Data Corporation (IDC) conducted research on their shipments showing 70.9 million shipments of tablets worldwide in 2011 and an estimated 117.1 million and 165.9 million in 2012 and 2013 respectively [15]. Thus, there is great opportunity for mHealth in using these mobile devices and, in fact, a significant number of mHealth applications have been already developed for these platforms.

Telecommunications technology aside, it is clear that there is still a long way to go in defeating illness. In 2008, WHO estimated a total of 56.8 million deaths and only 5.1 million of them were caused by injuries. The rest were caused by communicable disease, maternal and perinatal conditions and nutritional deficiencies (15.6 million deaths), and noncommunicable conditions (36.1 million deaths) [16]. Nevertheless, attention should be focused not only on the diseases that cause death, but also the diseases or conditions that can cause a disability or loss of health. In 2004, 2.9% of the world’s population were severely disabled, and 12.4% were moderately long-term disabled. In this context, it is essential to know the prevalence of an illness or condition, ie, the number of people who have the condition at any moment [17].

**Figure 1:**
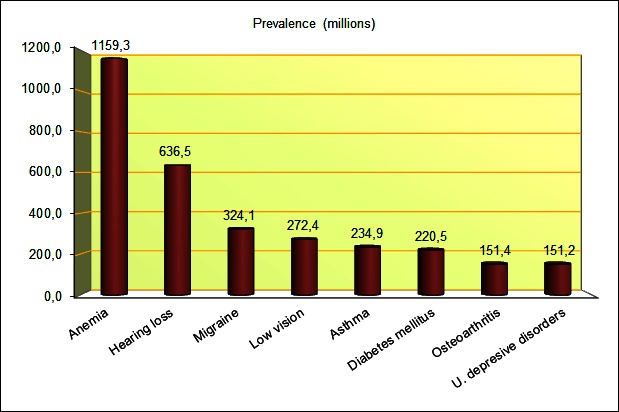
The 8 most prevalent conditions by the WHO.

According to WHO’s latest update (2004) of the Global Burden of Disease (GBD) [17], the most prevalent conditions are iron-deficiency anemia (IDA), hearing loss, migraine, low vision, asthma, diabetes mellitus, osteoarthritis (OA), and unipolar depressive disorder. The prevalence of each condition is shown in Figure 1. IDA represents 50% of the total cases of anemia (even though both terms are usually used interchangeably, they are not the same). The biggest percentage of affected can be found in underdeveloped and developing zones, in Africa, South East Asia, and Western Pacific, most of them women of reproductive age and children [18-20]. There are two types of hearing loss: moderate or greater hearing loss, which affect 275.7 million individuals, and mild hearing loss, with 360.8 million individuals [21-24]. Migraines are the most prevalent chronic neurological disorder in adults [25], with 11% of affected in Western countries [26-30].

The Global Data on visual impairment 2010 [31] indicates 246 million people with low vision and 39 million blind, equaling a total of 285 million people with any type of visual impairment [32-34]. Asthma is the most common chronic disease in childhood, and most asthma-related deaths take place in poor and developing countries [35-38]. It is estimated that 347 million people have diabetes mellitus [39], commonly named diabetes, but different from diabetes insipidus [40-55]. OA is the most prevalent musculoskeletal disease, and it is thought that 9.6% of men and 18% of women over 60 years have this condition [56-59]. Finally, there are more than 350 million individuals with any unipolar or bipolar depressive disorder [60-63].

To date, there are many published articles about types of wireless connections for mobile devices [8,9,64,65], articles about evaluations of apps for specific objectives [66-68], and reviews of apps of a determined device, software, or field [4,69,70], but there are not articles about the deadliest or the most prevalent conditions and diseases. Hence, the main aim of this paper is to study the existing applications for mobile devices exclusively dedicated to the eight most prevalent conditions [17] and to analyze a sample of the apps for each condition. The goal was to find the number of apps related to each condition, their common features, comparing the commercial ones with those used in research, and finding possible gaps in the development of these types of applications and whatever else might arise. For these purposes, a review has been done: (1) research of published articles containing specific target strings, obtained by search queries in a number of databases, and (2) research of applications related to these conditions in mobile phone application stores.

## Methods

Two different reviews were developed. The first was a literature review and the second, a commercial applications review. Both were current as of April 2013. Finally, one app for each review and each condition was chosen for an in-depth analysis and comparison.

### Literature Review

The literature review was developed on the following systems and databases: IEEE Xplore, Scopus, ScienceDirect, Web of Knowledge, and PubMed. After trying several combinations of words and expressions, the following combinations of terms were sought in the metadata field on each of these databases: “condition name” AND mobile AND (applications OR application OR apps OR app); m-health AND “condition name”; “mobile phone” AND “condition name”; smartphone AND “condition name”, where “condition name” is the name of each of the most prevalent conditions as they are issued by [17]. The results are limited to the last 10 years, from 2003 forward. The eligibility criteria were the following: publications not centered on applications using mobile phones or devices were dismissed, as were Web applications not optimized for mobile phone displays. Only papers published in English were studied. Papers on applications for several different diseases or conditions were rejected except for those with an important part of the application dedicated to the related condition. Some aspects were added due to the lack of sufficient results when executing some searches: instead of the search strings with “iron-deficiency anemia”, just “anemia” was used and the British term “anaemia”. Similarly, on IEEE Xplore, the search string with “anemia” was changed for the string anemia AND applications. Finally, the strings with “unipolar depressive disorders” were replaced by strings with “depressive disorder”, “depressive disorders”, “depressive episodes”, “depressive episode”, and “depression”.

### Commercial Apps Review

The second review, the study of the commercial applications, was carried out on the application stores of the most popular smartphones brands [14,71] which are, in descending order of market share, Google play of Google Android [72], Apple iTunes [73], BlackBerry World of BlackBerry (previously RIM or Research In Motion Limited) [74], Windows Phone Apps+Games Store of Microsoft [75], and Nokia’s Ovi Store [76].

For this research, the name of the condition was searched (eg, “hearing loss”) and the eligibility criteria used were the following: applications not centered on the specific condition, not in English, or those included in the category of games, entertainment, or music were dismissed, as well as applications designed for pets or animals, and applications that are actually journals or magazines about the condition. During the search, the following issues were faced:

- On iTunes, apps for iPod and iPhone were separate from the ones for iPad, hence only apps for the first ones were searched, excluding the apps exclusively designed for the Apple tablet.
- Instead of “iron-deficiency anemia”, the word searched was “anemia” due to the lack of results.
- The Ovi search engine does not handle search phrases nor logical operators such as “AND” or “OR” correctly; therefore, the results for “hearing loss” and “low vision” were totally distorted and these results were not considered. In the case of “diabetes mellitus”, no problems were presented because both words are very specific about the condition.
- In the case of diabetes mellitus, the strings “diabetes mellitus” and “diabetes” were used.
- For “unipolar depressive disorders”, instead of this string, “depressive disorders” and “depression” were used because of the absence of results.
- There were some issues with Google play: when searching for “depression” and “diabetes”, the store indicated at least 1000 results but showed only 480. Google was asked about this conflict and the issue is still under investigation, but it is assumed that the first number is the correct one (>1000). In other cases such as the search for anemia, a certain number of results had been found but while exploring the results pages, the last pages (usually between one and three) were blank. Consequently, the number indicated by the store was different from the number of apps shown. In these cases, the number of apps shown was the one used.
- In the case of depression and diabetes, only the first 20 applications were analyzed due to the number of results; therefore, there is potential for completely new investigative work.

### Eligibility Criteria for the In-Depth Analysis and Procedure

For the in-depth analysis, two applications were chosen for each condition: one obtained from the literature research and the other from the commercial apps review.

For the mobile applications found in publications, we decided to study the most recent paper on each condition taking into account only the year. In the case of two or more articles published in the same year, the final article was selected considering the impact factor of the journal where it is published (a conference article is considered at a lower level than journal articles), and its number of citations. If, again, two or more articles were published in the same journal and had the same number of citations, these papers were read and the most interesting according to the authors’ opinion was chosen. Articles about reviews of several apps or with insufficient information about the app were dismissed. If the app studied was available in stores, it was downloaded and personally tested on an iPhone 4 in the case of an iOS app or a Samsung Galaxy S SCL GT-I9003 in case of an Android app.

To evaluate the papers, after reading them individually, the authors convened to discuss opinions and fill in a table of features. For apps available on the market, one of the authors downloaded them for a joint evaluation at the meeting.

In the case of commercial apps, we preferred to choose apps from the same store for every condition and to use the store with the most extended software for smartphones. Therefore, Google play was selected because Android fulfilled this prerequisite [71]. For each condition, it was determined to opt for the first relevant free app with a rating, by users, of 3 or more stars, which Google play shows when searching by the condition sorted by popularity. In addition, another prerequisite was that the app had to be designed for patients, not for health care providers. This way, the most popular free app related to the condition with an evaluation over the mean and designed for the general public was analyzed. However, in the case of anemia, every result with a rating of 3 stars or more was aimed at caregivers; hence, we made an exception where the selected app was not intended for patients and did not fulfil all the previous requisites. The apps were tested on a Samsung Galaxy S SCL GT-I9003.

For the analysis of the commercial apps, the procedure followed was similar to the one developed with the research papers. The authors downloaded them on the mentioned mobile phone Samsung Galaxy S before meeting to study the apps together and complete the previously initiated table of features.

## Results

### Mobile Applications in Literature

The results of relevant papers on each database and each condition are shown in Table 1. The last row contains the number of different papers found for each condition.

Diabetes was the most investigated condition followed by asthma and depression. There is a significant gap between these three conditions and the rest since the next most investigated conditions were hearing loss and low vision with 9 different publications; this contrasts significantly with the 32 papers on depression. The order of the remaining conditions is: OA sixth, anemia seventh, and migraines last.

### Mobile Applications in Stores

The findings on the commercial apps review are shown in Table 2. It shows the number of relevant applications out of the total number of applications found in each store. In the case of diabetes mellitus and depression, the results obtained were separated by “diabetes mellitus” and “diabetes” in the first case, and “depressive disorders” and “depression” in the second case. The last row shows the addition of all the applications located in all the stores for each condition, but it is important to note that some of the applications designed for a specific system were also designed for other systems. Thus, an application developed for Apple iOS can also be developed for Android or Windows Phone, for example.

These results illustrate that the store with most apps is Google play, followed closely by iTunes. The rest of the stores have fewer apps for the conditions searched. Windows Phone Apps+Games Store is the third in number of applications, and it seems that Ovi Store has more applications than BlackBerry World, although that is not clear because of the malfunction of its searching engine mentioned in the Methods section.

Comparing the number of conditions, it is obvious that the conditions with more applications are diabetes and depression. After these two, migraine and asthma are equal with 112 apps, followed by low vision, hearing loss, OA, and finally anemia.

### Analysis of a Sample of Reference Apps

The papers and the commercial apps studied are summarized in Table 3 [77-97]. Figures 2 to 9 show snapshots from the commercial apps.

Tables 4 and 5 show the results from the analysis of some characteristics of the selected applications.

**Table 1:** Results of the literature review.

	Anemia	Hearing loss	Migraine	Low vision	Asthma	Diabetes mellitus	OA	Depression
IEEE	2	4	2	0	8	16	0	6
Scopus	3	8	2	7	29	112	6	22
ScienceDirect	0	0	0	1	1	5	1	5
WoK	1	5	1	3	25	79	5	18
PubMed	1	2	1	1	16	53	3	13
Total	5	9	3	9	36	140	6	32

**Table 2:** Results of the commercial apps review.

	Anemia	Hearing loss	Migraine	Low vision	Asthma	Diabetes		OA	Depression	
						Diabetes	Diabetes mellitus		Depression	Depressive disorders
Google Play	7/74	17/42	57/201	33/43	44/226	>1000	19/67	16/46	>1000	1/5
iTunes	7/21	32/37	46/102	30/46	57/124	605	17/21	5/16	419	0/0
BlackBerry	0/0	0/0	5/6	0/0	6/7	33	0/0	0/0	13	0/0
Windows	0/0	3/5	4/8	1/1	4/14	81	2/3	2/2	69	0/0
Ovi Store	0/0	-	0/0	-	1/2	40	15/40	1/1	35	-
Total	14	52	112	64	112	>1759	53	24	>1536	1

**Table 3:** Summary of the papers and commercial apps.

Health condition	Name of the paper/app	Description
Anemia	Activity and school attendance monitoring system for adolescents with Sickle cell disease [77]	Paper about the app SickleSAM, designed for Android, whose purpose is monitoring the school attendance of children affected by sickle cell disease, which usually causes anemia
	MD Series: Anemia - Free [78]	App for caregivers that provides some educational tools for the diagnosis and management of different types of anemia in adult patients
Hearing loss	Mobile software Apps support personalized-SRO and serial monitoring with results indicating early detection of hearing loss [79]	Article presenting a software application called OtoID used in a PDA connected to an audiometric unit for monitoring hearing change because of ototoxic medication and others factors, using a testing protocol called Sensitive Range for Ototoxicity (SRO)
	Hearing Tests [80]	App with a hearing test that uses sounds of different frequencies in order to check the user’s hearing
Migraine	From a traditional behavioral management program to an mHealth app: Lessons learned in developing mHealth apps for existing health care programs [81]	Application for behavioral migraine management for iPad called iBMM, which can be used for learning relaxing and pain management techniques, tracking migraine attacks and contacting a counselor
	My Headache Log Pro [82]	App for tracking headache attacks by creating a diary of them, with its triggers, symptoms and medications used, and it also allows emailing these notes to the doctor
Low vision	Crowdsourcing subjective fashion advice using VizWiz: Challenges and opportunities [83]	The authors use VizWiz [84] for assisting people with vision impairments in matching garments and being dressed in a fashion way, with the advice of some volunteers
	A.I.type EZReader Theme Pack [85]	Keyboard design for Android smartphones with big keys, high contrast, helpful colors, and audio aid, specially developed for people with visual problems
Asthma	Control of Allergic Rhinitis and Asthma Test (CARAT): dissemination and applications in primary care [86]	The authors talks about an app called m.Carat [87] developed for Android and iOS, consisting of several modules in which asthma and allergic rhinitis (ARA) patients can read news about ARA, record daily events and medications, quantify the level of control of their ARA, and note tasks or reminders
	SIGN Asthma Patient Guide [88]	Guidance for asthmatic patients and relatives in order to know and take control over their condition. It has a section dedicated to patients and other dedicated to parents or carers of asthmatic children
Diabetes	The development of an innovative mobile phone App for Type 1 diabetes alcohol education [89]	The authors develop the app for iOS and Android Type 1 diabetes friend: alcohol guide [90,91], which tries to educate young people with type 1 diabetes about alcohol, meeting clinical guidelines
	OnTrack Diabetes [92]	App for managing diabetes by tracking several data, such as blood glucose and pulse, medication, exercise, and weight
OA	PAGAS Portable and accurate gait analysis system [93]	Paper about a system called PAGAS (Portable and Accurate Gait Analysis System), which includes an Android app in a smartphone connected via Bluetooth to a sensor positioned on the foot and whose purpose is monitoring the gait of patients with altered gait because of different health conditons
	Osteoarthritis of knee [94]	App with information and animated exercises specially designed for osteoarthritis of knee
Depression	CBT for depression: a pilot RCT comparing mobile phone vs computer [95]	The authors use in their study the application VirtualClinic - The Get Happy Program [96] for iOS, which is a cognitive therapy intervention for the management of depression through a comic book story in which users will learn how to manage their depression
	Positive Thinking [97]	App that contains many quotes for helping depressed people and even allows users to write and share their own thoughts

**Table 4:** Analysis of features (Part 1) of the selected apps.

Condition	Name	Rating	Class	Internet requirement	Clinical/Non-clinical	Data visualization
Anemia	SickleSAM (not commercial)	-	Tracking	No	Clinical	Graphs
	MD Series: Anemia – Free	4.9	Educational	No	Both	Text
Hearing loss	OtoID (not commercial)	-	Diagnosis	No	Clinical	Text, graphs
	Hearing Tests	3	Diagnosis	No	Nonclinical	Text
Migraine	iBMM (not commercial)	-	Educational, guidelines, monitoring	Some functions	Both	Video, graphs, text
	My Headache Log Pro	4.1	Monitoring	Only for sending mails	Both	Text, graphs
Low vision	VizWiz	4.5	Assistive	Yes	Nonclinical	Photos, text, audio
	EZReader Theme Pack	4.4	Assistive	No	Nonclinical	Text, audio
Asthma	m.Carat	-	Monitoring, assistive	Some functions	Nonclinical	Graphs, text, pictures
	SIGN Asthma Patient Guide	4.9	Informative, guidelines	Some functions	Nonclinical	Text, pictures
Diabetes mellitus	Type 1 diabetes friend: alcohol guide	-	Educational, informative	Some sections	Nonclinical	Text, photos
	OnTrack Diabetes	4.5	Monitoring	Only for sending mails	Both	Text, graphs
Osteoarthritis	PAGAS (not commercial)	-	Medical results	No	Both	Text, graphs
	Osteoarthritis of knee	5	Treatment	No	Nonclinical	Text, video
Depression	VirtualClinic – The Get Happy Program	-	Educational, guidelines	Unknown	Nonclinical	Pictures, comic, text
	Positive thinking	4.3	Kind of treatment	Some functions	Nonclinical	Text

**Table 5:** Analysis of features (Part 2) of the selected apps.

Name	Context awareness	Therapist intervention	Interaction with users	Frequency of use	Interface	Public
SickleSAM (not commercial)	User, location	Yes	No	Continuous	Simple	School children
MD Series: Anemia – Free	No	-	No	Occasional	Not intuitive	Anemia specialists
OtoID (not commercial)	Ambient noise	Yes	No	Regular	Basic	Ear specialists
Hearing Tests	No	No	No	Occasional	Basic	General
iBMM (not commercial)	No	Yes	No	Frequency of migraine attacks	Not intuitive	General
My Headache Log Pro	Preferences, location	Possible	No	Frequency of migraine attacks	Complex, several functions	General
VizWiz	Location	No	Yes, several ways	Frequent	Simple	General
EZReader Theme Pack	Language	No	No	Every time keyboard is used	Basic	General
m.Carat	User, preferences, language	No	No	Constant	Complex	General
SIGN Asthma Patient Guide	No	No	No	Occasional	Basic	General
Type 1 diabetes friend: alcohol guide	No	No	No	Occasional	Simple	Young people (aged 18-21)
OnTrack Diabetes	Preferences	Possible	No	Several times per day	Normal, several functions	General
PAGAS app (not commercial)	Sensor	Possible	No	Regular	Basic	General
Osteoarthritis of knee	No	No	No	Frequent	Simple	General
VirtualClinic – The Get Happy Program	Unknown	No	No	Regular	Simple	General
Positive thinking	Preferences	No	No	Regular	Basic	General

**Figure 2:**
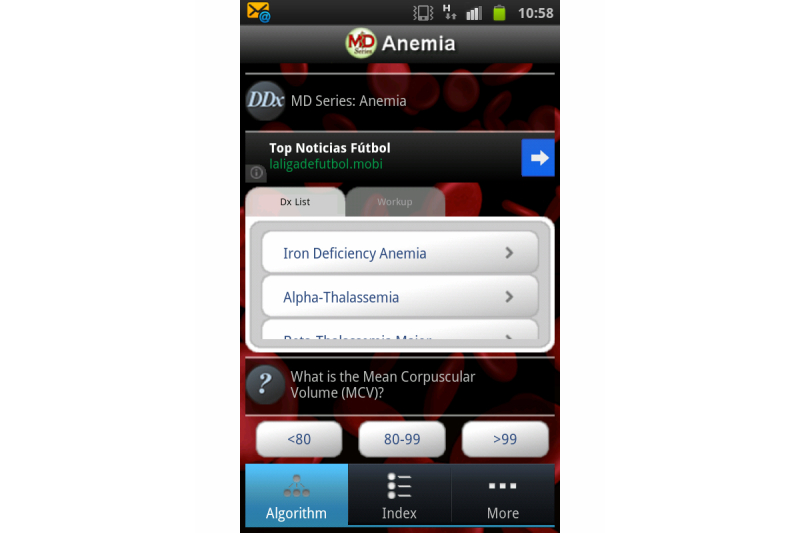
Snapshot of MD Series: Anemia–Free.

**Figure 3:**
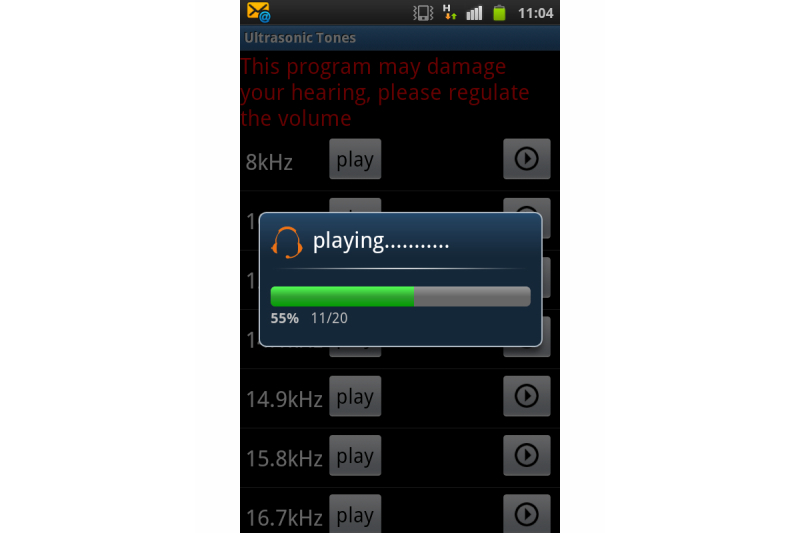
Snapshot of Hearing Tests.

**Figure 4:**
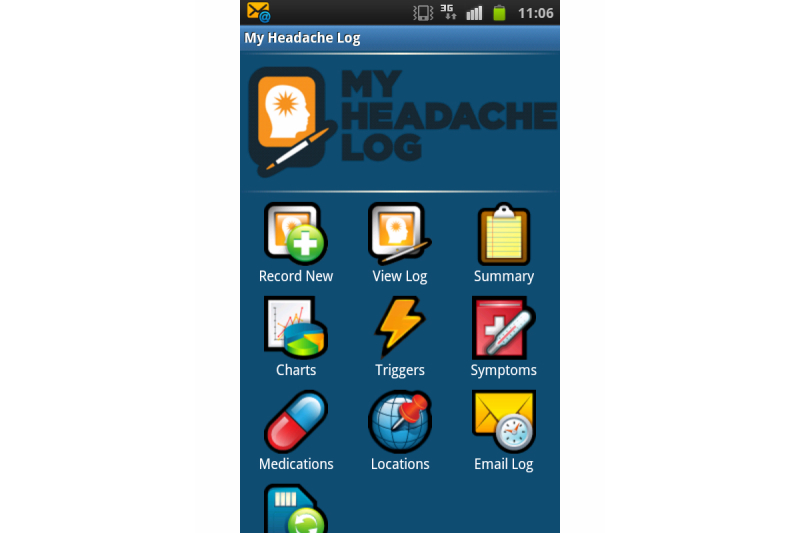
Snapshot of My Headache Log Pro.

**Figure 5:**
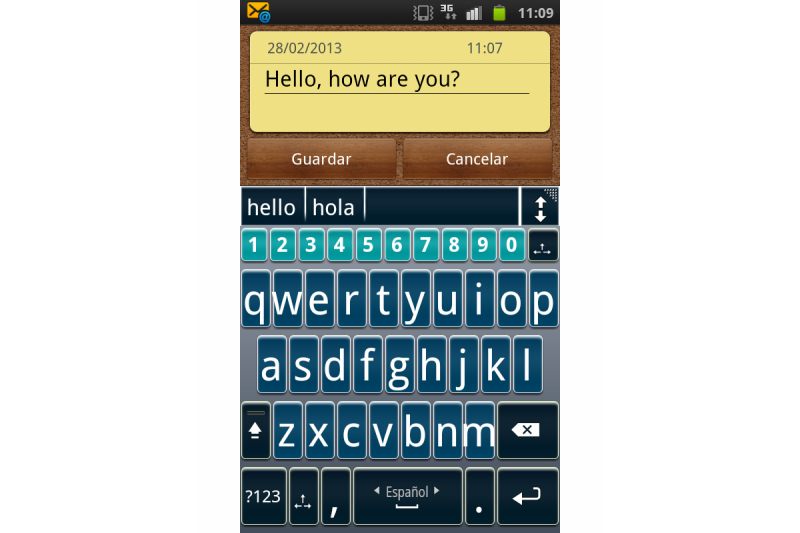
Snapshot of AI type EZReader Theme Pack.

**Figure 6:**
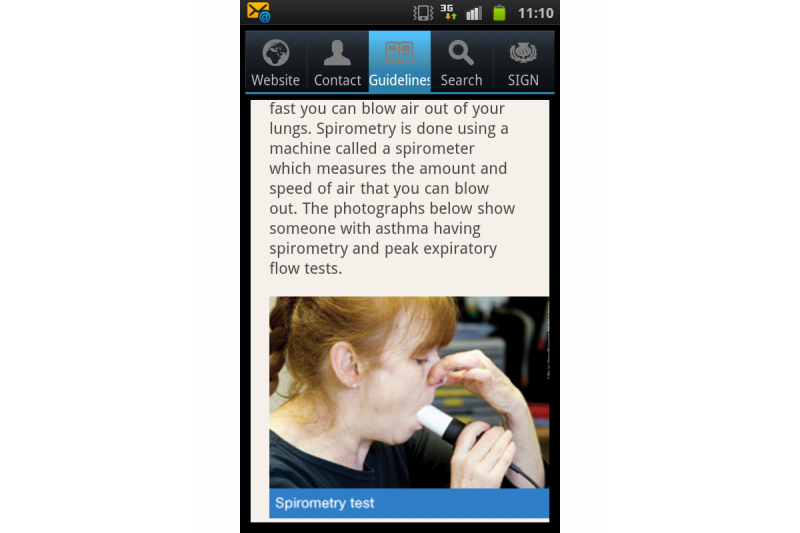
Snapshot of SIGN Asthma Patient Guide.

**Figure 7:**
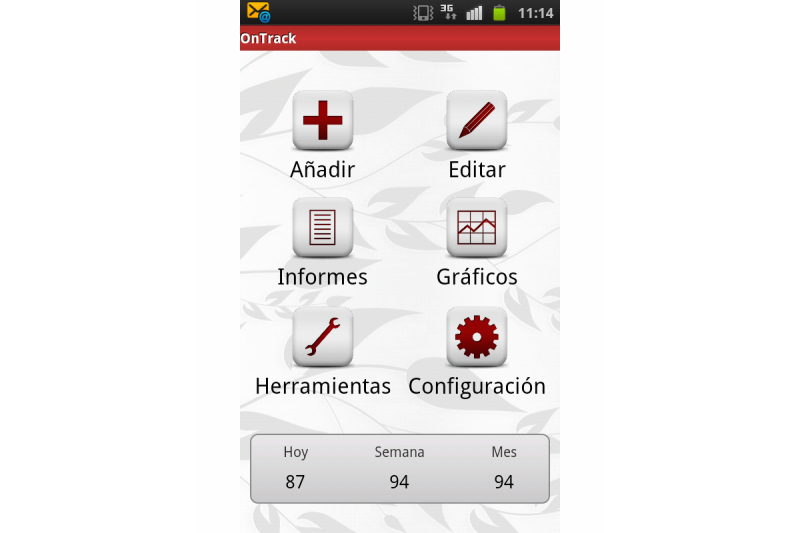
Snapshot of OnTrack Diabetes.

**Figure 8:**
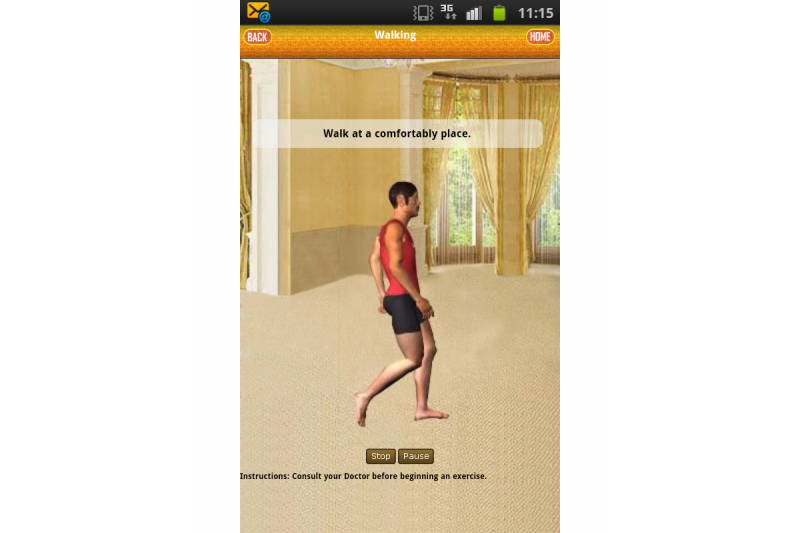
Snapshot of Osteoarthritis of knee.

**Figure 9:**
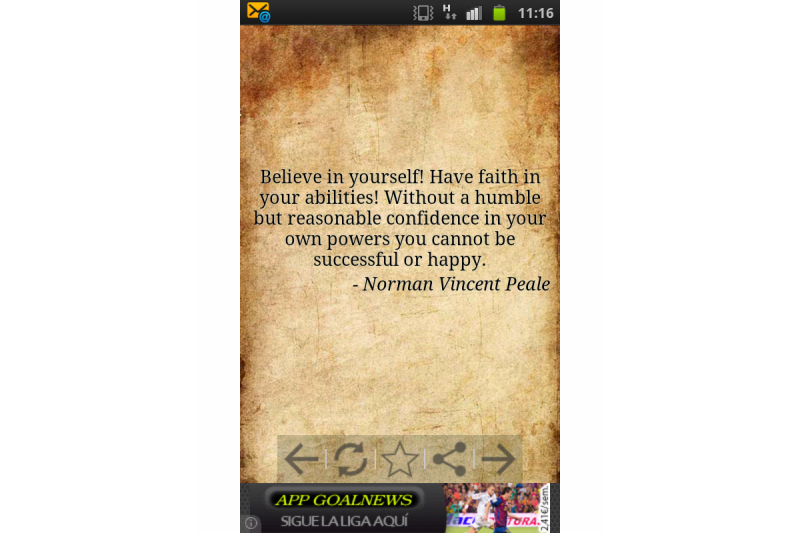
Snapshot of Positive Thinking.

## Discussion

The results of the literature and the commercial reviews show several interesting conclusions. First, the literature review indicates that there are few results compared to the commercial review. This means that the development of mHealth apps has a commercial and economic motivation more than a research motivation. Maybe it would be better to merge both, ie, first developing an app while investigating and then using it for commercial purposes.

Another important finding is the difference in the number of mobile applications for different conditions. There are conditions with more than 1000 apps, such as diabetes or depression while there are others with a range between 14 and 112 apps. The most prevalent condition (anemia) is surprisingly the second with less research and the first with less commercial apps, while two of the less prevalent (diabetes and depression) are the object of a huge number of applications and research. This may be because the majority of IDA cases are located in underdeveloped or developing countries [17,18] where smartphones or tablets are not as widely spread as in the developed countries, and therefore, it is not worthwhile developing apps for IDA. Meanwhile, diabetes or unipolar depression are common conditions associated with modern lifestyles typical of developed zones where there is a strong social conscience of these conditions. However, this is not borne out when comparing the numbers of those affected by diabetes or depression and by IDA in developed countries, because IDA is more extended in these zones than the others. Hence, the probable explanation for this lack of apps for IDA is that the social conscience of it is much less than that of the others and, therefore, it is underinvestigated. For this reason, in light of the numbers, it could be profitable and worthwhile creating apps for IDA.

Following the previous finding, the four most prevalent conditions have fewer apps and research than the remaining four, excluding OA. In addition, there is an important social factor for some of the most prevalent conditions, such as hearing loss or low vision. Therefore, it seems that there is a lack in the research on apps for these conditions and an opportunity for developers.

Contrasting the literature review with the commercial review, it is worth highlighting the cases of asthma and migraines. In the literature, asthma has more results than depression, but it has far fewer results in the commercial review. The opposite occurs with migraines, which move from the last position in number of literature results to the third position in the commercial review. Therefore, there is more work done on commercial apps for migraines than in research applications, as opposed to the situation for asthma.

Finally, comparing the number of apps available on each store, it is clear that application developers prefer Android and iOS for their projects, followed distantly by Windows Phone. Ovi Store and BlackBerry World suffer from a significant lack of apps, which means that developers are not interested in these markets. This is somewhat surprising in the case of BlackBerry since it is the third smartphone platform in market share [71]; hence, there may be an opportunity for developers to fill this empty space in BlackBerry’s app store.

Determined criteria were followed in order to select the latest apps published in literature and the most popular and best valued by users’ apps in commercial stores. The goal was to choose a sample of reference apps to analyze. These findings are given below.

The majority of apps are for monitoring, assistance, or informing about the condition. In general, apps for diabetes, migraines, and asthma are designed for monitoring the condition, and many have informative sections (or stand-alone informative apps). Apps for low vision are principally assistive, and the aim of most apps for depression is to raise the mood of the affected in several ways. But there are also informative and educational apps, which cover the other conditions, except for anemia, for almost every app is aimed for professional caregivers. For OA, there are an equal number of educational tools and apps with remedies or exercises for managing the pain, and for hearing loss the apps are divided between apps for hearing checks and informative apps.

Table 4 shows that for most apps, the Internet is not required or only required for some functions such as sending emails, which is useful for circumstances or situations where an Internet connection is not available. However, there are apps where an Internet connection is required, eg, for VizWiz. Typically the apps are not designed only for clinical purposes; proof of this is that only some apps developed for research are intended for clinical use. Others can have nonclinical and clinical use (always with the consent of the professional caregiver), but the majority have nonclinical purposes. In addition, there is a link between clinical apps and therapist intervention: if the app is clinical, the therapist intervention is needed. If the app is applicable for both clinical and nonclinical use, the therapist intervention is possible, but not obligatory. With respect to users’ interaction, most of the apps do not have this functionality and work as stand-alone apps. Only those conditions that can be relieved in some way with the collaboration of other affected individuals or a determined community of users, such as depression or diabetes, have apps with modules used for these communications.

According to the results, the preferred method of data visualization is text, followed by graphs and pictures or photos. Whereas text visualization is used in almost every case, the use of graphs is common in monitoring or tracking applications in order to show data in a more comfortable and visual way. Something similar occurs with pictures and videos, which are normally used in apps with educational or informational purposes, while audio is a typical aid of low vision apps and is also used for hearing tests in hearing loss apps. Apps with only text visualization or with text and pictures have generally a basic or simple interface, whereas those with graphs or more than two visualization types have a more complex interface, not necessarily intuitive at first use. It is not shocking for apps with several types of data visualization to have a complex interface with several functions, but it is surprising that some of them have such unintuitive ones. Developers need to be careful when designing the interface and its use.

Generally there is no relation between the type of apps and the types of context awareness. Only location awareness can be linked to tracking and monitoring apps where it is important to state the place where an event occurs. For frequency of use, monitoring and assistive apps have a continuous or very frequent use, depending in some cases, such as migraine or asthma, on the frequency of the attacks. The same happens with apps designed to provide some kind of treatment. On the other hand, educational or informative apps are used more infrequently. It might be a good idea to merge monitoring and educational tools in the same app in order to improve frequency of use, number of users, and economic profit, but always with special attention to the interface design. Finally, the majority of the apps analyzed are aimed for the general public who are affected by each condition, which is logical because usually developers do not want to reduce their user circle.

For future work there are various paths to take. It is necessary to fill the lack of anemia apps by creating one aimed for patients, first educational and informative and then exploring other possible opportunities. Another field to populate is related to low vision and hearing loss apps because, as said before, there are few compared with other less prevalent conditions. Nevertheless, in this case it is important to carefully select the intended user because most people with severe hearing loss or low vision problems are over 60 years old in developed countries and do not usually use a smartphone [98]. Hence, designing an assistive app for a 16-50 year-old deaf/blind (or with a severe disability) target group that typically has smartphones can be very useful for the user and even profitable for the developers. In addition to this, the possibilities for creating assistive apps in these fields are enormous.

## Abbreviations

ARA: asthma and allergic rhinitis
CARAT: Control of Allergic Rhinitis and Asthma Test
CBT: cognitive behavioral therapy
GBD: global burden of disease
GOe: Global Observatory for eHealth
HSPA: High-Speed Packet Access
IDA: iron-deficiency anemia
IDC: International Data Corporation
OA: osteoarthritis
PAGAS: Portable and Accurate Gait Analysis System
PDA: personal digital assistant
RCT: randomized controlled trial
RFID: Radiofrequency Identification
RIM: Research In Motion Limited
SIGN: Scottish Intercollegiate Guidelines Network
SRO: sensitive range for ototoxicity
WBAN: Wireless Body Area Network
WHO: World Health Organization
WiMAX: Worldwide Interoperability for Microwave Access
WLAN: Wireless Local Area Network
WPAN: Wireless Personal Area Network
WSN: Wireless Sensor Networks
